# Simple non-invasive in vivo measurement of stress difference in the skin

**DOI:** 10.1007/s10237-026-02087-1

**Published:** 2026-06-05

**Authors:** Hannah Conroy Broderick, Wenting Shu, Aisling Ní Annaidh, Michel Destrade

**Affiliations:** 1 School of Mechanical and Materials Engineering, University College Dublin, Belfield, Dublin 4, Ireland; 2 School of Mathematical and Statistical Sciences, University of Galway, University Road, Galway, Ireland; 3 Present Address: School of Engineering, University of Leicester, Leicester, UK

**Keywords:** Skin mechanical properties, In vivo testing, Surface wave propagation, Finite element simulations

## Abstract

Accurate, non-invasive quantification of in vivo skin stress would improve surgical planning and objective assessment of skin mechanics. We present a simple acousto-elastic approach that estimates the in vivo in-plane stress difference from surface wave speeds measured along two orthogonal directions on the skin. For a large class of incompressible hyperelastic anisotropic models with one family of parallel fibres aligned with the skin tension lines, we show that the principal Cauchy stress difference satisfies: $\sigma _1 - \sigma _2 \simeq \rho (v_\text {max}^2 - v_\text {min}^2)$, where $\rho$ is the tissue mass density, and $v_\text {max}$, $v_\text {min}$ are the Rayleigh wave speeds along, and orthogonal to, the direction of greatest tension, respectively, with a relative error below 9 percent. We validate the formula with finite element simulations in plane strain using the neo-Hookean and Holzapfel-Gasser-Ogden materials. In the neo-Hookean case, stress differences calculated from simulated wave speeds agree with the ground truth within 2.3 to 7.9 percent across pre-stretches $\lambda _1 = 1.05$ to 1.20. In the anisotropic case with fibres parallel to the principal pre-stress, the error is 0.25 to 2.81 percent over the same range. These results provide a proof of concept for estimating the in-plane stress difference in skin from orthogonal Rayleigh-wave speeds, within the assumptions of a homogeneous fibre-reinforced hyperelastic model.

**Supplementary Information:**

The online version contains supplementary material available at 10.1007/s10237-026-02087-1.

## Introduction

The human skin is a complex material that is known to be (mostly) under tension in vivo. Surgeons currently rely on qualitative techniques such as pinch tests and palpations when seeking to evaluate the skin tension. These techniques can only give subjective estimates of the in vivo stress and depend on the experience of the surgeon (Lackmann et al. 2023). Tension is also the main factor that influences healing outcomes, with reducing non-physiological tension a priority during wound healing (Parikh et al. 2022). Quantitative knowledge of the in vivo stress in skin would aid in preoperative reconstructive surgery planning, e.g., by providing safe limits of skin stress, predicting scar formation, or accurately estimating the area of skin required for defect repairs. Here, we propose a method using simple acoustic measurements, which relies on an acousto-elastic modelling framework.

Like most soft tissues, skin has an inherent residual stress in vivo (Alexander and Cook 1977; Holzapfel 2005; Flynn and McCormack 2010). It results from growth and remodelling mechanisms (Rodriguez et al. 1994) and is, in general, very complex (Holzapfel 2001; Ciarletta et al. 2016). The level of residual stress can have a significant effect on material parameter estimation, and yet accurate quantitative data on the level of in vivo residual stress is still lacking (although some progress has recently been made for hard elastic solids such as steel (Li et al. 2020) and for thin isotropic films (Li et al. 2022)).

Skin tension lines are used by surgeons in preoperative planning to determine the best location and orientation for an incision. By making incisions along these skin tension lines, which are parallel to the maximal skin tension (Borges 1984), surgeons reduce scarring and the chance of infection (Parikh et al. 2022). There is little understanding, however, of the underlying structural contributions or residual stresses related to this effect. It is only recently that the orientation of collagen fibres was related to the direction of skin tension lines (Deroy et al. 2017). Further work on mechanical characterisation of the skin is needed to better understand these effects.

Determining the underlying tension in the skin has thus far mainly been focused on the orientation of skin tension lines. Some proposed in vivo testing methods are similar to those commonly used for determining material properties, including tension (Flynn et al. 2010; Paul et al. 2016) and suction (Laiacona et al. 2019; Song et al. 2022). However, since the skin tension lines are related to the orientation of the collagen network, a method that *does not* deform this network would more accurately represent the in vivo state.

Recent efforts have focused on acoustic techniques to characterise the skin, including its mechanical properties (Kirby et al. 2022; Luo et al. 2015; Pitre et al. 2019; Feng et al. 2022) and the orientation of skin tension lines (Nagle et al. 2023; Deroy et al. 2017; Elouneg et al. 2025), but there has been little work in quantifying the in vivo stress in skin. Reihsner et al. (1995) excised skin from cadavers and used biaxial stretching to stretch the sample to its original size, measuring the resulting tension. They also showed that the in vivo stress is anisotropic. This measurement is likely not representative of the in vivo setting, due to the differences between living and preserved non-living tissue (Joodaki and Panzer 2018). As regards in vivo methods, Diridollou et al. (2000) and Flynn et al. (2011) both treated the pre-stress as a parameter and fitted it to their experimental data. Diridollou et al. (2000) used a suction-based method and predicted an “average” pre-stress value, which does not account for the inherent anisotropy in the skin and the stress. Flynn et al. (2011) used a tensiometer, which deforms the collagen network, and they then fitted the data to an isotropic model. Lackmann et al. (2023) measured forces in dog cadavers using by creating wounds of increasing sizes. Recently, Nagle et al. (2024) used a Gaussian process model trained on finite element simulations to predict the stress and pre-stretch of skin using measurements of surface wave speeds, namely the Rayleigh and Supershear wave speeds. However, this method did not include the anisotropy of the skin and has yet to be robustly validated against experimental data. An in vivo, non-destructive, non-invasive testing method that quantifies the pre-stress in the skin and takes into account both material and stress anisotropy has yet to be proposed.

Here, we use an acoustic surface wave based technique to measure the in vivo stress difference in skin, i.e. the difference in stress magnitude along the two principal directions. We derive an expression relating the in vivo stress difference in two orthogonal directions to the surface wave velocity (Sect. 2.1). This relationship is valid when the pre-stretch is aligned with the fibres and for a large class of material hyperelastic models, where the strain energy is the sum of an isotropic neo-Hookean term and a generic convex function of the first anisotropic invariant $I_4$ (one family of parallel fibres). Other than these assumptions, our expression assumes no mechanical properties (only the mass density of the skin), and so the in vivo stress difference can be measured independently of a specific (hyperelastic) material model. We then validate this expression using Finite Element (FE) simulations (Sect. 2.2).

We show that the maximal (minimal) in vivo stress is related to the fastest (slowest) speed of the measured surface Rayleigh wave and that our expression is accurate within a 9% error when compared to the measured stress difference. The finite element simulations highlight the feasability of the method, where the error between calculated and actual stress difference confirms that it is within the range predicted by the analytical model.

In the present study, this validation is performed numerically within the same idealised constitutive and geometric assumptions used in the derivation, rather than in a fully physiological model of skin.

## Methods

### Analytical modelling

We model the region of interest in the skin as a semi-infinite, homogeneous, hyperelastic, anisotropic, incompressible material subject to a large homogeneous pre-strain, resulting from the application of a uniform pre-stress. We take the skin tension lines (defined as the lines of greatest tension in the body) to be aligned with a family of parallel fibres.

We assume that the material’s strain energy density *W* follows the following class of functions,

1 $$\begin{aligned} W = \frac{\mu }{2}\left( I_1 - 3\right) + f(I_4), \end{aligned}$$

where $\mu$ is the infinitesimal shear modulus of the isotropic matrix (it is equal to *E*/3, where *E* is the infinitesimal Young modulus in the absence of fibres), $I_1 = \text {tr}\ \boldsymbol{C}$ is the first principal invariant of $\boldsymbol{C}$ (the right Cauchy-Green deformation tensor), and *f* is a yet unspecified convex function of the anisotropic invariant $I_{4}  = M \cdot CM$, where $M$ is the initial orientation of the fibres, such that $f(1)=f'(1)=0$. This model includes the *standard reinforcing model* (Merodio and Ogden 2002),

2 $$\begin{aligned} W = \frac{\mu }{2}\left( I_1 - 3\right) + \frac{\gamma }{4}(I_4-1)^2, \end{aligned}$$

where $\gamma >0$ is a measure of the fibre stiffness. We take $2\gamma >3\mu$ to ensure that the fibres are stiffer than the matrix in the small uniaxial deformation regime (see the appendix). It also includes the *Holzapfel-Gasser-Ogden model* (HGO) with one family of fibres (Holzapfel et al. 2000),

3 $$\begin{aligned} W = \frac{\mu }{2}\left( I_1 - 3\right) + \frac{\gamma }{4 k}\left[ \text{ e}^{k(I_4-1)^2} - 1\right] , \end{aligned}$$

where $k>0$ is a stiffening parameter (for *k* small, Eq. (2) is recovered).

Here, the usual $2\gamma > 3\mu$ condition is introduced as a sufficient small-strain uniaxial criterion ensuring that the fibre contribution dominates the isotropic matrix response. We do not claim that this threshold is unique under all multiaxial loading paths, and stricter conditions may arise in particular loading specialisations.

Next we use Lamb’s result (Lamb 1904) about the far-field wavefront generated by a point-load impact, namely that it propagates at the speed of a (Rayleigh) surface wave. We call $v_\text {max}$ and $v_\text {min}$ the extreme values of that speed, taking place along, and at right-angle to, the skin tension lines. Then, a straightforward analysis, detailed in the appendix, reveals that $\sigma _1 - \sigma _2 = \rho (v^2_\text {max} - v^2_\text {min}) + \mu \varepsilon$, where $|\varepsilon | < 0.0874$, irrespective of the choice of the function *f* in the model (1). It follows that, taking $(\sigma _1-\sigma _2)/\mu$ as a non-dimensional measure of the stress difference, the formula

4 $$\begin{aligned} \sigma _1 - \sigma _2 = \rho (v^2_\text {max} - v^2_\text {min}) \end{aligned}$$

is valid within an error of less than 9%.

### Finite Element simulations

We validate the equation (4) with FE Simulations using Abaqus/Standard and Abaqus/Explicit. We test both an isotropic neo-Hookean model (nH) ($f(I_4)=0$ in (1)) and an anisotropic Holzapfel-Gasser-Ogden (HGO) model with one family of fibres (3) (Holzapfel et al. 2000) The fibres in the anisotropic model are initially aligned along the $x_1$-direction.

The parameters for both models are listed in Table 1. For the isotropic part we take the value for $\mu$ from Ní Annaidh et al. (2012) based on uniaxial tensile tests on human skin. For the anisotropic model we use the one-fibre-family HGO form as implemented in Abaqus, with Abaqus parameters $k_1$ and $k_2$ corresponding to the parameters in (3) through $k_1=\gamma /2$ and $k_2=k$. Ní Annaidh et al. (2012) reported $k_1 = 245.3$ kPa, but this value led to very strongly elongated wavefronts in our wave-propagation simulations, which reduced the usefulness of the directional comparison for the present validation study. We therefore used the reduced value $k_1 = 24.53$ kPa, which retains a clear anisotropic response while allowing more informative comparison of the propagating wavefronts. We take $k_2$ sufficiently small so that the HGO response remains close to that of the standard reinforcing model (2) over the deformation range considered here, while retaining the Abaqus implementation of the HGO law. Additionally, we specify the initial Poisson ratio as $\nu =0.495$, as simulations using Abaqus/Explicit require some compressibility, and noting that the default Abaqus value of 0.475 allows for too much departure from the near perfect incompressibility of biological soft tissues and may introduce modelling innacuracy (Destrade et al. 2012).

**Table 1:** Material parameters and dimensions used in FE simulations. The shear modulus $\mu$ (kPa), material density $\rho$ ($\text {kg/m}^{3}$) and Poisson’s ratio $\nu$ are the same for both models. The HGO model in Abaqus has two additional material parameters: a measure of the fibre stiffness $k_1$ (kPa), and a stiffnening parameter $k_2$. The sample has side length *L* (mm) and thickness *H* (mm)

	$\mu$ (kPa)	$\rho$ ($\text {kg/m}^{3}$)	$k_1$ (kPa)	$k_2$	$\nu$	*L* (mm)	*H* (mm)
neo-Hookean	201.4	1100	–	–	0.495	30	5
HGO	201.4	1100	2453	0.0001	0.495	40	5

**Fig. 1:**
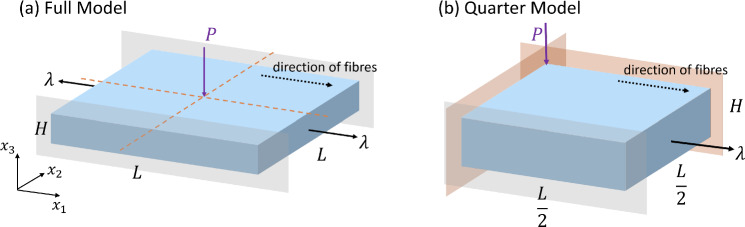
Boundary Conditions of the FE Simulations. **a** The full model with side length *L* and thickness *H*. A pre-stress is induced in the sample by a stretch $\lambda _1=\lambda$ in the $x_1$-direction (along the fibres) while the sample is prevented from expanding in the $x_2$-direction by lubricated fixed rigid plates (grey shaded planes), so that $\lambda _2=1.0$. An instantaneous pressure $P=100$ kPa is applied at the centre of the upper face. **b** The quarter model is simulated by exploiting the symmetry indicated by dashed lines in (a). Symmetry conditions are applied on the relevant faces

We model the skin as a homogeneous rectangular block undergoing a pre-stress (Fig. 1). We are interested in measuring the wave characteristics in a small range, say up to 5 mm from the impact site, but we simulate a larger sample to avoid the effects of the wave reflecting off the boundaries (see dimensions in Table 1). Note that the dimensions for the HGO model are larger than the nH model, so to better avoid these reflection effects, as the stiffer fibres present in the HGO material lead to faster waves (see Sect. 3).

We use the symmetry of the system and model one quarter of the sample, with symmetry boundary conditions on the appropriate faces.

To induce a pre-stress in the skin, we impose a plane-strain pre-deformation during the Abaqus/Standard step, in the sense that the $x_2-$direction is fixed kinematically, so that $\lambda _2 = 1.0$, while four different levels of pre-stretch are applied in the $x_1-$direction, namely $\lambda _1 = 1.05, 1.10, 1.15, 1.20$. In addition, because the relevant boundary is traction-free in the thickness direction, the out-of-plane normal stress satisfies $\sigma _{33} = 0$.

Note that the pre-stretch is parallel to the initial fibre orientation in the HGO model (see Fig. 1), as in in vivo human skin (Deroy et al. 2017).

The resulting stress state and nodal deformations from Abaqus/Standard are imported into Abaqus/Explicit for the dynamic analysis (Fig. 1).

This two-stage procedure was adopted as the most efficient procedure to simulate a static analysis (applying pre-stress) followed by the dynamic analysis (wave propagation).

We also apply encastré conditions on the bottom face of the sample to ensure that the wave propagates during the dynamic analysis. The wave is generated via an applied pressure on a small surface (radius $\approx 0.1$ mm) at the centre of the stressed sample for a short time. Here, we applied a 100 kPa pressure for 10 $\upmu$s at the centre of the sample. After the pressure is removed, we track the resulting wave propagating for 500 $\upmu$s.

The mesh must be chosen appropriately so that the simulation converges; here we chose a mesh size that is approximately 1/15th of the surface wave wavelength (see for instance Li et al. (2020)). For the isotropic model simulations, a mesh size of $8 \times 10^{-5}$ $\text {m}$ with about 1080k elements meets this criterion for all cases. To reduce computational cost, a coarser mesh (size $=3.75 \times 10^{-4}$ $\text {m}$) was used far from the region of interest. For the anisotropic model simulations, a mesh size of $1.6\times 10^{-4}$ $\text {m}$ with about 490k elements was used to meet the criterion for the $\lambda =1.05, 1.10, 1.15$ cases. This was chosen as a compromise between computational cost and wave resolution. However, due to the increase in wave velocity, a finer mesh was needed for the $\lambda =1.20$ case to meet the 1/15th of the surface wave wavelength criterion. For this case, a mesh size of $1.1\times 10^{-4}$ with about 1490k elements was used. All cases were meshed with 3D 8-node rectangular elements (C3D8).

**Fig. 2:**
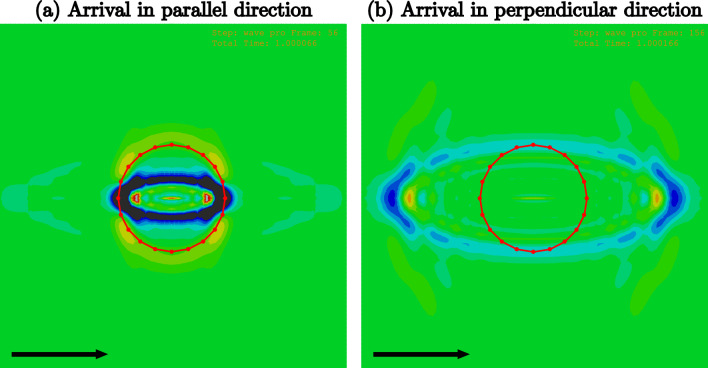
The Rayleigh wave propagating for HGO simulations with pre-stretch $\lambda =1.10$, in the $x_1$-$x_2$ plane on the surface of the material, zoomed in to an approximately 12 mm square to focus on the area of interest. The arrow indicates the direction of the fibres and maximal pre-stress and the circle is 2 mm from the centre of the impact. The contours represent the displacement in the $x_3$-direction (out of plane) and have range from approximately $-0.52$ (dark blue) to $0.27 \mu \text {m}$ (red) (see Fig 4). **a** Arrival of the Rayleigh wave parallel to fibres and the maximal in vivo stress at $\sim 66$ $\upmu \text {s}$. **b** Arrival of the Rayleigh wave in the direction perpendicular to the fibres and maximal pre-stress at $\sim 166$ $\upmu \text {s}$

We measure the wave velocity by tracking the arrival time of the first significant wave at two measurement points (see Fig. 2).

**Fig. 3:**
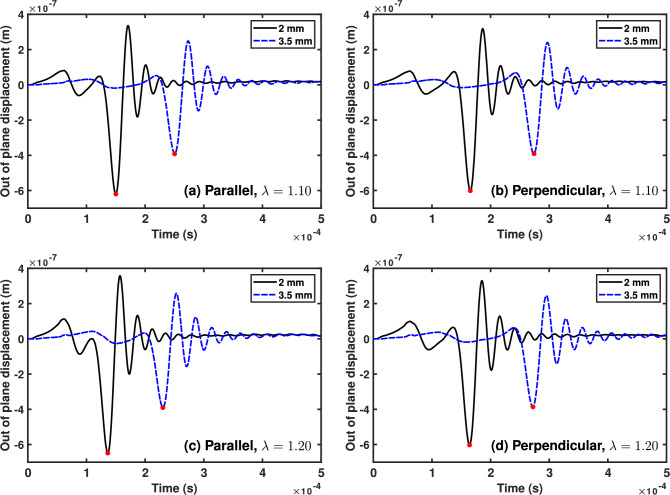
Out of plane displacement 2 mm (solid/black) and 3.5 mm (dashed/blue) from the centre of the impact, from FE simulations using a pre-stressed neo-Hookean model. The dot indicates the time at which the wave arrives at the relevant in plane location. **a** Parallel to the maximal pre-stress with pre-stretch $\lambda =1.10$, **b** Perpendicular to the maximal pre-stress with pre-stretch $\lambda =1.10$, **c** Parallel with $\lambda =1.20$, and **d** Perpendicular with $\lambda =1.20$

**Fig. 4:**
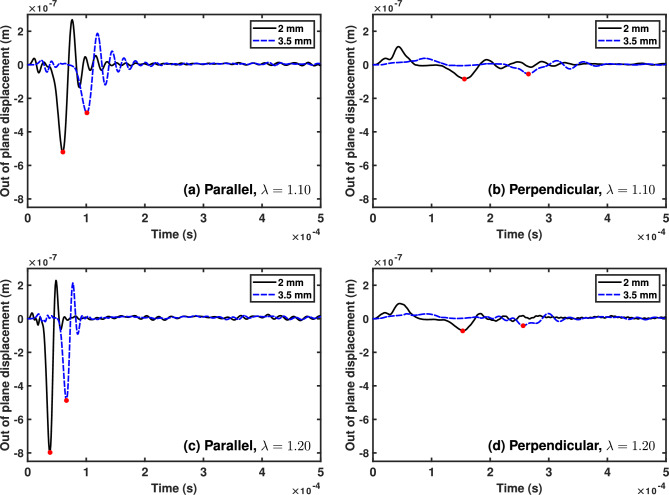
The out of plane displacement at 2 mm (solid/black) and 3.5 mm (dashed/blue) for the FE simulations using the HGO model. The red point indicates the arrival time of the wave at the relevant location and in the relevant direction (**a**) Parallel to the fibres and the direction of applied pre-stress, with pre-stretch $\lambda =1.10$, (**b**) Perpendicular to the fibres and direction of applied pre-stress with pre-stretch $\lambda =1.10$, (**c**) Parallel with $\lambda =1.20$ and (**d**) Perpendicular with $\lambda =1.20$

Here, we measured the out of plane displacement at 2 and 3.5 mm from the centre of the sample and tracked the minimum corresponding to the Rayleigh wave at each point (see Figs 3, 4 in Sect. 3). We selected these values because the points should be far enough away from the impact to avoid spurious oscillations due to the impact, but close enough to the impact to avoid interference from reflections at the boundaries. Additionally, the distance between the points should not be too large, to avoid errors due to wave attenuation, or so small that the faster waves cannot be measured (e.g. at higher stresses with the HGO model).

## Results

The simulations show that there is more than one type of wave propagating in the material as a result of the impact. On the surface, the first wave observed is a supershear wave, a shear wave with a high speed and low amplitude (see for instance Nagle et al. (2024)). The Rayleigh wave is the ellipsoidal front that follows, indicating that it is fastest along the principal pre-stress and fibres. The simulations also highlight that the Rayleigh wave is the highest amplitude wave generated, and thus the easiest to measure experimentally.

An example of the displacement contours for the HGO model with a pre-stretch $\lambda =1.10$ is given in Fig. 2. The circle indicates the distance 2 mm from the centre of the sample, i.e. the impact zone. The wave reaches the 2 mm circle first in the parallel direction at $\sim 66 \upmu {s}$ (Fig 2a) and later arrives perpendicular to the fibres and maximal pre-stress at $\sim 166 \upmu {s}$ (Fig 2b).

We extract the out of plane displacement 2 mm and 3.5 mm from the centre, in both the parallel and perpendicular directions (i.e. maximal and minimal speeds) at each level of stress. The Rayleigh wave is identifiable by the global minimum in all cases. We then calculate the time taken to travel this known distance, and calculate the Rayleigh wave velocity. We note that the out of plane displacement *cannot* be used to calculate the velocity directly, as the Rayleigh wave propagates *in plane*. Here, we identify the wave by the propagation of the wave front as seen on the surface (Fig. 2).

In Fig. 3, we plot this out of plane displacement for the (initially isotropic) nH model for two stress cases, $\lambda =1.10$ and $\lambda =1.20$. The highlighted point indicates when the wave arrives at the specified location and direction. We calculate the Rayleigh wave velocity in both directions using these plots, and then calculate the stress difference $\Delta \sigma _\text {cal}$ predicted by equation (4). The actual stress difference $\Delta \sigma _\text {sim}$ can be determined from the simulations. The velocities, stress differences, and resulting errors calculated from the nH simulations are given in Table 2. The Rayleigh velocity is always larger in the parallel direction (direction of applied stress), and increases as the pre-stress increases. The error between the simulated and calculated stress difference is below the 9% threshold determined by the analytical model (Sect. 2.1) in all four cases, indicating the relevance of the proposed formula in this simple case.

**Table 2:** Summary of neo-Hookean FE Simulations. The simulated stress difference $\Delta \sigma _{\text {sim}}$ (kPa), simulated Rayleigh velocities parallel and perpendicular to the principal stress $v_{\parallel ,\perp }$, stress difference calculated from the simulated Rayleigh velocities using Eq (4) $\Delta \sigma _{\text {cal}}$, and the resulting percentage error between the actual simulated and calculated stress difference for different levels of initial pre-stretch

$\lambda$	$v_{\parallel }$ (m/s)	$v_{\perp }$ (m/s)	$\Delta \sigma _{\text {sim}}$ (kPa)	$\Delta \sigma _{\text {cal}}$ (kPa)	% Error
1.05	13.25	12.55	20.61	19.74	4.22
1.10	14.02	12.61	42.16	41.20	2.27
1.15	14.67	12.69	64.65	59.55	7.88
1.20	15.35	12.69	88.12	82.08	6.85

We then move to validating the formula in the anisotropic HGO case, which includes both a pre-stress and aligned fibres. We plot the out-of-plane displacement for HGO simulations with pre-stretch $\lambda =1.10$ and $\lambda =1.20$ in Fig. 4. Here, the difference between the wave speeds and amplitudes in the parallel and perpendicular direction is much larger, so care must be taken when determining the features related the the Rayleigh wave. We see from the simulations that the global minimum still represents the Rayleigh wave, although it is more difficult to identify in Figs. 4b,d. The time for the wave to travel between 2 and 3.5 mm is shorter in the parallel direction, indicating that the wave is faster in that direction. The plots also show that the Rayleigh wave speed increases as the pre-stress increases. We again use the calculated velocities to calculate the stress difference $\Delta \sigma _\text {cal}$ with equation (4), the results of which are given in Table 3. The Rayleigh wave speed again increases in the principal direction as the principal pre-stress increases. Additionally, the presence of fibres that are stiffer than the surrounding matrix significantly increases the Rayleigh wave velocity in the direction of the fibres, as expected. Finally, the error between the stress difference calculated according to equation (4) and that measured from the simulations is less than the $9\%$ limit determined by the analytical model.

**Table 3:** Summary of HGO FE Simulations. The simulated stress difference $\Delta \sigma _{\text {sim}}$ (kPa), simulated Rayleigh velocities parallel and perpendicular to the principal stress and fibres $v_{\parallel ,\perp }$, stress difference calculated from the simulated Rayleigh velocities using Eq (4) $\Delta \sigma _{\text {cal}}$, and the resulting percentage error between the simulated and calculated stress difference for different levels of initial pre-stretch

$\lambda$	$v_{\parallel }$ (m/s)	$v_{\perp }$ (m/s)	$\Delta \sigma _{\text {sim}}$ (kPa)	$\Delta \sigma _{\text {cal}}$ (kPa)	% Error
1.05	25.19	12.67	528.56	521.53	1.33
1.10	34.42	13.00	1150.02	1117.70	2.81
1.15	42.65	12.38	1863.44	1832.40	1.67
1.20	51.22	14.01	2663.72	2670.42	0.25

These two sets of simulations highlight the feasibility of the proposed formula (4). In practice, the Rayleigh wave velocities can be easily measured in vivo non-invasively (see for instance Nagle et al. (2023)), and the stress difference can be calculated using the formula. We note that the plane strain conditions imposed in the simulations may be different to those in skin in vivo (Reihsner et al. 1995). However, both in-plane stresses were tensile in the simulations, as in the biaxial conditions of the formula.

## Discussion

### Principal findings and significance

We show that the difference between the two in plane principal stresses can be estimated directly from a pair of Rayleigh wave speeds, without prior identification of constitutive parameters. The relation $\sigma _1 - \sigma _2 \simeq \rho (v_\text {max}^2 - v_\text {min}^2)$ follows from acousto-elasticity under broad assumptions on the strain energy, and finite element analysis support its accuracy. Errors stay below $9\%$ for neo-Hookean materials and below $3\%$ for anisotropic HGO materials with fibres aligned to skin tension lines.

The reported differences between the proposed analytical solution and the directly measured finite element solution reflect a combination of wave-picking uncertainty, discretisation and transient effects. We also note that the larger directional separation of wave speeds in the anisotropic case may in some cases make the arrival times easier to identify, which could help explain why the anisotropic errors appear to be lower.

The results suggest a possible route towards quantifying stress anisotropy in vivo with minimal instrumentation, which could inform incision orientation, safe closure limits, flap and graft design, and prediction of scar risk where high residual tension persists. At present, however, this study should be viewed as a proof of concept within an idealised modelling framework. Further validation in more physiologically realistic computational models and in experiments will be needed before quantitative clinical use can be established.

### Limitations

The analysis assumes a semi-infinite, homogeneous, incompressible body with one dominant fibre family aligned with the lines of tension, and the finite element validation was intentionally performed within the same idealised framework. Real skin is thin, layered, heterogeneous, viscoelastic, residually stressed, and attached to subcutaneous tissues. Accordingly, when the acoustic wavelength is not small compared with tissue thickness or structural length scales, departures from the semi-infinite homogeneous assumption may alter the measured Rayleigh-wave speeds.

In addition, the FE validation uses a single representative material parameter set for each constitutive model. A broader parametric exploration would be useful to assess the robustness of the proposed relation more systematically.

Finally, the wave identification relies on singling out the Rayleigh wave arrival from surface signals, which may be complicated by overlapping transient waves, shear waves, attenuation, and noise. The current validation is numerical, with idealised loading and boundary conditions.

**Fig. 5:**
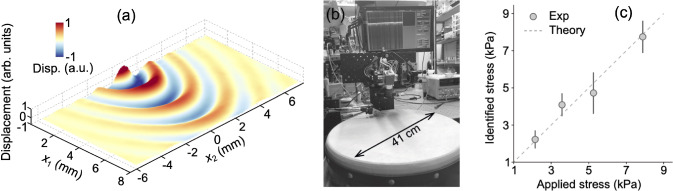
Several protocols allow for the measurement of the speed of elastic waves on the surface of soft matter. (**a**): The 3D wave field generated by a spherical probe hitting a thin rubber membrane, as captured by Optical Coherence Tomography. The initial membrane’s dimensions were $40\times 16 \times 0.5$ mm; its initial shear modulus was $\sim 180$ kPa and it was stretched in the $x_1$ direction by a 200 kPa stress. The vertical displacements were of the order of 0.1 $\mu$m and are magnified to an arbitrary unit to be visible. The color scale for these displacements goes from $-1$ (blue) to $+1$ (red) (Li et al. 2022). (**b**): A similar experiment, generating Lamb waves on the stretched sheep skin of a *Bohrán*, an Irish traditional drum. (**c**): For bulk (body) shear waves propagating inside a muscle with one preferred direction of parallel fibres, a formula linking wave speeds to stress gives excellent agreement (Zhang et al. 2023)

In reality, biological skin has many characteristics which are not captured by the simple model (1) such as viscoelasticity, inhomogeneities, residual stresses, fibre misalignment or multiple fibre families, etc., and which will complicate greatly the computation of the Rayleigh wave velocities $v_\text {max}$ and $v_\text {min}$. We nonetheless note that the stress difference equation (4) has also been established and validated computationally and experimentally for bulk waves in pre-stressed steel (Li et al. 2020) and soft tissues (Zhang et al. 2023) and for Lamb waves in animal skin (Li et al. 2022), see Fig. 5, so that it might prove robust to further refinements.

### Future work

Immediate future work should concentrate on experimental validation via synthetic skin surrogate materials under controlled boundary conditions. Building on this, controlled in vivo studies, for example on the forearm, can be used to measure the Rayleigh wave velocities, $v_\text {max}$ and $v_\text {min}$, with sensor, optical or ultrasound based surface wave elastography (see examples in Fig. 5). The validity of the resulting stress difference can then be tested against reference methods such as tensiometers and compression-based devices.

Alongside these experiments, the modelling framework can be extended to layered, viscoelastic, and heterogeneous skins, to quantify thickness and depth-dependent structure effects on the Rayleigh relation, and update the error bounds accordingly if needed.

A further direction is to move from estimating only the stress difference to recovering the full in-plane stress field by combining multiple propagation directions. It would turn the method into a tool for complete, quantitative preoperative stress mapping.

## Supplementary Information

Below is the link to the electronic supplementary material.

Supplementary file 1 (mp4 905 KB)

Supplementary file 2 (mp4 798 KB)

## Figures and Tables

**Fig. 6:**
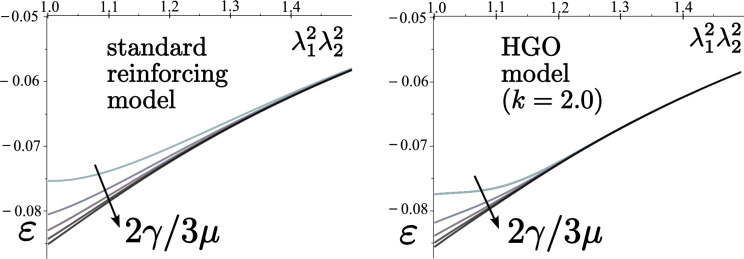
The formula $\sigma _1-\sigma _2 = \rho (v^2_\text {max} - v^2_\text {min})$ is valid within a relative error $\varepsilon$ of less than 9% when skin is modelled by any neo-Hookean reinforcing model. Relative error for increasing ratios of fibre-to-matrix infinitesimal stiffnesses $2\gamma /3\mu = 2, 3, \ldots ,6$, as indicated by the arrow. Left: standard reinforcing model. Right: HGO model with stiffness parameter $k=2.0$, which is an extreme value, much larger than expected for skin

## Data Availability

No datasets were generated or analysed during the current study.

## Appendix: Stress formula from surface wave speeds

Here, as in the main text, we model the region of interest in the skin as being made of a semi-infinite, homogeneous, hyperelastic, anisotropic, incompressible material subject to a large homogeneous pre-strain, resulting from the application of a uniform pre-stress. We call $\rho$ its constant mass density. We assume that the greatest tension is aligned with a family of parallel fibres (Alexander and Cook 1977; Deroy et al. 2017).

We call $\boldsymbol{\sigma }$ the Cauchy (true) stress measure of this pre-stress, with $\sigma _1$ and $\sigma _2$ the largest and least principal components in the plane of the skin, while $\sigma _3 =0$ in the direction normal to the skin (free surface). We assume that the material’s strain energy density *W* has the form,

5 $$\begin{aligned} W = \frac{\mu }{2}\left( I_1 - 3\right) + f(I_4), \end{aligned}$$

where $\mu$ is the infinitesimal shear modulus of the isotropic matrix, $I_1 = \text {tr}\ \boldsymbol{C}$ is the first principal invariant of $\boldsymbol{C}$ (the right Cauchy-Green deformation tensor), and *f* is a yet unspecified convex function of the anisotropic invariant $I_4$ (here $I_4 = C_{11}$), such that $f(1)=f'(1)=0$.

Consider that the solid is homogeneously deformed along the Eulerian principal axes $(x_1,x_2,x_3)$ so that it occupies the $x_3 \ge 0$ region. Once the large pre-deformation has taken place, the principal axes of stress are aligned with the principal axes of strain, and the principal stresses are related to the principal stretches through (Merodio and Ogden 2002)

6 $$\begin{aligned} \sigma _1 = -p + \mu \lambda _1^2 + 2f' \lambda _1^2, \qquad \sigma _2 = -p + \mu \lambda _2^2, \qquad \sigma _3 = -p + \mu \lambda _3^2, \end{aligned}$$

where *p* is a Lagrange multiplier introduced by the constraint of incompressibility, $\lambda _1$ is the principal stretch in the direction of greatest tension, also the direction of the fibres, $\lambda _2$ is the in-plane stretch in the direction at right angle to the direction of greatest tension, $\lambda _3$ is the in-depth stretch, and $\lambda _1\lambda _2\lambda _3=1$ by incompressibility.

Specialising now to uni-axial tension in the direction of the fibres (direction of the skin tension lines): $\sigma _1=\sigma >0$, $\sigma _2=\sigma _3=0$, leads to an equi-biaxial deformation, with $\lambda _1=\lambda$, $\lambda _2=\lambda _3=\lambda ^{-1/2}$, by symmetry and incompressibility. Then $I_4=\lambda ^2$ and, solving for *p*, we find

7 $$\begin{aligned} \sigma = \mu \left( \lambda ^2 - \lambda ^{-1}\right) + 2f' \lambda ^2, \end{aligned}$$

where the prime denotes the derivative with respect to $I_4$.

In the infinitesimal regime, $\lambda = 1+e$ where $|e|\ll 1$ is the elongation, and at first order, this equation gives $\sigma = 3\mu e + 4 f''(1)e$, confirming that the ratio $2\gamma /3\mu$ for the parameters of the neo-Hookean reinforcing model (1) and of the HGO model (3) measures the relative stiffness of the fibres compared to the matrix.

In the case of biaxial tension (as in in vivo skin (Reihsner et al. 1995)), with $\sigma _1$ applied along the direction of the fibres to stretch them by $\lambda _1$, $\sigma _2$ applied at right-angle to the fibres with corresponding stretch ratio $\lambda _2$, and the surface being free of traction so that $\sigma _3=0$, we find that

8 $$\begin{aligned} \sigma _1 = \mu \left( \lambda _1^2 - \lambda _1^{-2}\lambda _2^{-2}\right) + 2f' \lambda _1^2, \qquad \sigma _2 = \mu \left( \lambda _2^2 - \lambda _1^{-2}\lambda _2^{-2}\right) . \end{aligned}$$

A Rayleigh surface wave travels along the $x_1$-direction with speed $v_1$ given by $\rho v_1^2 = \gamma _{13} - \eta _1^2\gamma _{31}$, where $\eta _1$ is the root of the cubic (Destrade et al. 2005)

9 $$\begin{aligned} \eta ^3 + \eta ^2 + \frac{2\beta _{13}+2\gamma _{31} - \gamma _{13}}{\gamma _{31}}\eta - 1 =0. \end{aligned}$$

Similarly, a surface wave travels along the $x_2$-direction with speed $v_2$ given by $\rho v_2^2 = \gamma _{23} - \eta _2^2\gamma _{32}$, where $\eta _2$ is the root of the cubic Eq. (9) with the index 1 replaced by the index 2. Here

10 $$\begin{aligned} \gamma _{ij} = \mathcal {A}_{0ijij}, \qquad \beta _{ij} = (\mathcal {A}_{0iiii} + \mathcal {A}_{0jjjj})/2 - \mathcal {A}_{0iijj} - \mathcal {A}_{0ijji}, \end{aligned}$$

where $\mathcal {A}_{0jilk}$ are the instantaneous elastic moduli. These equations are valid when the principal axes of the Cauchy stress are aligned with those of Eulerian strain, which is the case for an initially isotropic material, and also for anisotropic solids when the fibres are aligned with those directions, as here. It is also the case for in vivo human skin (Deroy et al. 2017).

Now, calling $\lambda _i$ the principal stretches, we have

11 $$\begin{aligned} \boldsymbol{F} = \text {diag}(\lambda _1, \lambda _2, \lambda _3), \qquad I_1 = \lambda _1^2 + \lambda _2^2 + \lambda _3^2, \qquad I_4 = \lambda _1^2, \end{aligned}$$

where $\boldsymbol{F}$ is the deformation gradient and $\lambda _1\lambda _2\lambda _3=1$ due to incompressibility. For this model (Merodio and Ogden 2002; Destrade et al. 2008),

12 $$\begin{aligned} \mathcal {A}_{0jilk} = \mu \delta _{ik}B_{jl} + 2 f'\delta _{ik}m_jm_l + 4 f''m_im_jm_km_l, \end{aligned}$$

where $\boldsymbol{B} = \vec {FF}^T$ is the left Cauchy-Green deformation tensor and $\boldsymbol{m}$ is the stretch vector along the fibres (here $m_i = \lambda _1\delta _{i1}$). Hence we find

13 $$\begin{aligned}&\mathcal {A}_{01111} = \mu \lambda _1^2 + 2 f'\lambda _1^2 + 4f''\lambda _1^4, &  \mathcal {A}_{01313} = \mu \lambda _1^2 + 2 f'\lambda _1^2, \nonumber \\&\mathcal {A}_{03131} = \mathcal {A}_{03333} = \mu \lambda _3^2 = \mu \lambda _1^{-2}\lambda _2^{-2}, &  \mathcal {A}_{01133} = \mathcal {A}_{01331} = 0. \end{aligned}$$

We then find that $2\beta _{13} = \gamma _{13} + \gamma _{31} + 4 f''\lambda _1^4$ and the cubic (9) reduces to

14 $$\begin{aligned} \eta ^3 + \eta ^2 + \left( 3 + 4 \frac{f''}{\mu } {\lambda _1^6}{\lambda _2^2}\right) \eta - 1 =0. \end{aligned}$$

Similarly we find

15 $$\begin{aligned}&\mathcal {A}_{02121} = \mathcal {A}_{02323} = \mathcal {A}_{02222} = \mu \lambda _2^2, \qquad \mathcal {A}_{03232} = \mathcal {A}_{03333} = \mu \lambda _3^2, \qquad \mathcal {A}_{03322} = \mathcal {A}_{02332} = 0, \end{aligned}$$

and then $2\beta _{32} = \gamma _{23} + \gamma _{32}$ so that, in this case, the cubic (9) reduces to

16 $$\begin{aligned} \eta ^3 + \eta ^2 + 3\eta - 1 =0. \end{aligned}$$

The real root of this latter cubic is

17 $$\begin{aligned} \eta _0 = \frac{(26+6 \sqrt{33})^{1/3}}{3} - \frac{8}{3(26 + 6\sqrt{33})^{1/3}} - \frac{1}{3} \simeq 0.2956, \end{aligned}$$

and the root of the cubic Eq. (14) is

18 $$\begin{aligned} \eta = \frac{\nu ^{1/3}}{6} - \frac{2(8/3 + x)}{\nu ^{1/3}} - {\frac{1}{3}}, \quad \text {where} \quad \nu = 208+36\,x + 12\, \sqrt{528 + 360x + 105x^2 + 12x^3}, \end{aligned}$$

and $x = 4 (f''/\mu ) \lambda _1^6\lambda _2^{2}$. Notice that $x>0$ because *f* is convex. Plotting $\eta$ against *x* shows that it is a decreasing function, starting at $\eta _0$ and tending to zero in the $x\rightarrow \infty$ limit. Hence we establish the bounds $0<|\eta |<\eta _0$, which hold for any reinforcing model (1), irrespective of the actual form of *f* and of the pre-stress.

Now the speeds along $x_1$ and $x_2$ are given by

19 $$\begin{aligned} \rho v_\text {1}^2 = \mu \lambda _1^2 + 2f' \lambda _1^2 - \eta _1^2 \mu \lambda _1^{-2}\lambda _2^{-2}, \qquad \rho v_\text {2}^2 = \mu \lambda _2^{2} - \eta _0^2 \mu \lambda _1^{-2}\lambda _2^{-2}, \end{aligned}$$

and the combination of Eqs. (8) and (19) yields the stress difference as

20 $$\begin{aligned} \sigma _1 - \sigma _2 = \rho (v^2_1 - v^2_2) + \mu \varepsilon , \qquad \text {where} \qquad \varepsilon = (\eta ^2-\eta _0^2)\lambda _1^{-2}\lambda _2^{-2}. \end{aligned}$$

It follows that the formula $\sigma _1 - \sigma _2 = \rho (v_1^2-v_2^2)$ is valid up to a relative error of $|\eta ^2 - \eta _0^2|\lambda _1^{-2} \lambda _2^{-2}$. The skin is under in-plane extension strain ($\lambda _1>1$, $\lambda _2\ge 1$); it follows that $\varepsilon$, according to the bonds on $\eta$, is always less than $\eta _0^2 = 0.0874$. It is important to note that in our simulations we only considered the $\lambda _2=1$ or plane strain case. However, this leads to tensile in plane stresses ($\sigma _1$ and $\sigma _2$), as required by the analytical model and as in in vivo skin (Reihsner et al. 1995).

Figure 6 shows how $\varepsilon$ varies with $\lambda _1^2\lambda _2^2>1$ for the standard reinforcing model (2) and for the HGO model (3). For this latter example we take $k=2.0$ which is an extreme value, as typically *k* is less than 1.0 (see Ní Annaidh et al. (2012) for example, where it was estimated as $k = 0.1327$ for the human skin.)
